# Genome wide expression analysis of circular RNAs in mammary epithelial cells of cattle revealed difference in milk synthesis

**DOI:** 10.7717/peerj.13029

**Published:** 2022-03-01

**Authors:** Syed Mudasir Ahmad, Basharat Bhat, Zainab Manzoor, Mashooq Ahmad Dar, Qamar Taban, Eveline M. Ibeagha-Awemu, Nadeem Shabir, Mohd Isfaqul Hussain, Riaz A. Shah, Nazir A. Ganai

**Affiliations:** 1 Division of Animal Biotechnology, Faculty of Veterinary Sciences, Sher-e-Kashmir University of Agricultural Sciences and Technology of Kashmir, India, Srinagar, Jammu and Kashmir, India; 2 Department of Clinical Biochemistry, University of Kashmir, Srinagar, Jammu and Kashmir, India; 3 Sherbrooke Research and Development Centre, Agriculture and Agri-Food Canada, Sherbrooke, Canada; 4 Division of Veterinary Microbiology, SKUAST-Kashmir, Srinagar, Jammu and Kashmir, India

**Keywords:** CircRNA, Mammary gland, Bovine, RNA-Seq, Identification, Differential expression

## Abstract

Milk is an excellent source of nutrients for humans. Therefore, in order to enhance the quality and production of milk in cattle, it is interesting to examine the underlying mechanisms. A number of new investigations and research have found that, circRNA; a specific class of non-coding RNAs, is linked with the development of mammary gland and lactation. In the present study, genome wide identification and expression of the circRNAs in mammary epithelial cells of two distinct cattle breeds viz Jersey and Kashmiri at peak lactation was conducted. We reported 1554 and 1286 circRNA in Jersey and Kashmiri cattle, respectively, with 21 circRNAs being differentially expressed in the two breeds. The developmental genes of the established differentially expressed circRNAs were found to be largely enriched in antioxidant activity, progesterone, estradiol, lipid, growth hormone, and drug response. Certain pathways like MAPK, IP3K and immune response pathways were found significantly enriched in KEGG analysis. These results add to our understanding of the controlling mechanisms connected with the lactation process, as well as the function of circRNAs in bovine milk synthesis. Additionally, the comparative analysis of differentially expressed circRNAs showed significant conservation across different species.

## Introduction

Milk is a highly nutritious liquid produced in mammary glands of mammals to support and feed their new born babies during their initial months of life. Milk is secreted by epithelial alveolar cells that are organized into isolated pockets called lobules. Nutritional value of milk is very high and contains almost every nutrient that our body requires. Cow’s milk is a rich and economical source of proteins (casein and whey proteins), casein alone accounts for approximately 80% of milk protein. It is also a good source of fat, carbohydrates, hormones, vitamins and minerals (Elzoghby, El-Fotoh & Elgindy, 2011).

As milk production is an economic trait in dairy animals, an increase in the milk synthesis is a desirable goal for the dairy industry (Bionaz, Hurley & Loor, 2012). A deeper understanding of the factors controlling milk synthesis can therefore provide guidance on the methods and processes to enhance milk production. As reported earlier, hormones, protein factors and non-coding RNAs (ncRNAs) play a significant role in controlling the milk production (Bionaz, Hurley & Loor, 2012; Sandhu et al., 2016; Hansji et al., 2014).

Circular RNAs (circRNAs), discovered more than three decades ago (Hsu & Coca-Prados, 1997), have gained substantial attention in the last couple of years because of their potential function in eukaryotic cells. CircRNAs are one of the ncRNAs that are typically produced when pre-mRNA (pre-messenger RNA) is alternatively spliced which involves joining of upstream splice acceptor with downstream splice donor in a process called backsplicing forming a unique covalently closed circular structure lacking 3′ and 5′ ends (Morris & Mattick, 2014; Barrett, Wang & Salzman , 2015). This mechanism protects it from RNA exonucleases, stabilising it and making it less prone to degradation (Schindewolf, Braun & Domdey, 1996; Jeck & Sharpless, 2014; Memczak et al., 2013). CircRNAs contain exonic or intronic sequences of its parental genes and range in size from 100 nt to 4 kb (Szabo & Salzman, 2016). Mainly, three types of circRNAs have been identified: exonic circular RNAs (ecircRNAs), intronic circular RNAs (ciRNAs) and exon-intron circular RNAs (ElciRNAs). With the advances in RNA sequencing (RNA-Seq) technologies and bioinformatics, diverse properties of circRNAs are being explored and have been found to be expressed in all cells and tissues just like linear mRNAs (Mumtaz et al., 2020). Some circRNAs contain micro RNA response elements (MREs) that serve as competitive endogenous RNA and play a special regulatory role by functioning as micro RNA (miRNA) sponge, thereby controlling the expression of parent genes in nucleus (Zhang et al., 2013). Studies also suggest that circRNAs act as miRNA sponge and thus are capable of enhancing the target gene expression (Hansen et al., 2013). Certain circRNAs build protein complexes, regulate cell cycle and protein translation (Du et al., 2016). CircRNAs also function as protein scaffolds particularly for RNA-binding proteins (RBPs) including RNA Polymerase II (Zwierzchowski, 2005) and Argonaute (AGO) proteins. Moreover, circRNAs are involved in disease pathogenesis by directly regulating certain relevant pathways and maintaining mRNA homeostasis. Because of their vital role in cellular regulation, protein signaling and disease pathogenesis, circRNAs are becoming a new research hotspots in RNA biology (Liu et al., 2020).

Accounting the universal expression characteristics of circRNAs, these molecules have been found to be involved in the milk synthesis as well as mammary gland development of various species, like sheep (Wang et al., 2021; Hao et al., 2020a; Hao et al., 2020b), cattle (Zhang et al., 2016a; Zhang et al., 2016b), goat (Ma et al., 2019) and rat (Zhang et al., 2015). In the present study we performed a systematic examination and expression profile of circRNA by RNA-Seq in mammary gland epithelial cells (MECs) at peak lactation in two cattle breeds with distinct milk production properties *i.e.,* high lactating Jersey and low lactating Kashmiri cattle. We describe the type, length and chromosomal distribution of the circRNAs and their functional enrichment analysis with the aim to understand their role in lactation of dairy cows.

## Material and Methods

As a global trend, we concluded that the larger replicate numbers increase the power of differential expression analysis, except for low-expressed genes. We utilized RnaSeqSampleSize v2.0.0 (Zhao et al., 2018) for the estimation of samples required for the analysis. RnaSeqSampleSize recommended five samples (biological replicates) per condition for the identification of majority of diffentially expressed circRNAs.

In this study, a total of eight samples (four Jersey and four Kashmiri cows) at the peak lactation was collected from our published data in the SRA database (accession ID. SRR6324372), including one sample from each breed as technical replicate. The details of experiments followed are mentioned below.

### Sampling and isolation of MECs

Clean and fresh milk samples (1.5 l/cow) were collected from healthy and unrelated cows of each cattle breed by hand milking. The animals were kept in free stall sheds, provided with a complete healthy nutritious and balanced diet, and had unrestricted access to clean water and grass. The detailed sample collection including sample preparation and RNA sequencing were explained in the previous study (Bhat et al., 2020).

### Quantification of circRNAs

A total of eight samples (4 Jersey and 4 Kashmiri) at peak stage of lactation were selected for the identification of circRNAs mediating different milk traits in both breeds. The FASTQC program v0.11.2 was used to perform quality control on all raw reads (Chanumolu, Albahrani & Otu, 2019). The Cutadapt software v2.10 (Martin, 2011) and Sickle tool v1.33 (Joshi & Fass, 2011) were used to filter out adapter sequences as well as decreased and low quality reads. Using the HISAT2 program v2.03, the filtered reads were mapped to the reference bovine genome (ARS-UCD1.2) (Kim et al., 2019).

### Identification of differentially expressed circRNAs

Two-way approach was employed for the identification of differentially expressed (DE) circRNAs. At first seekCRIT tool v1.0.0.b (Chaabane et al., 2020) were run on filtered reads to identify DE circRNAs. Another strategy of identification of DE circRNA involves quantification of circRNAs (using CIRI and CIRC-explorer) and running edgeR on the quantified dataset.

Based on the expression of circular - RNAs, the quasi-likelihood F-test method of the edgeR program (McCarthy, Chen & Smyth, 2012) was used for identifying circRNAs that are differentially expressed between two breeds. Differentially expressed circRNAs between two contrast groups were screened using *P*-value <0.05 and logFC >1 threshold. The final set of DE circRNAs contains circRNAs common between seekCRIT dataset and edgeR dataset. CircRNAs whose log2FC >1 and *p*-value <0.05 were considered as DE in two breeds. To identify the conserved circRNAs, all DE circRNAs were compared against the known circRNAs from *Homo sapiens*, *Mus musculus*, *Sus scrofa*, *Gallus gallus* and *Canis lupus*.

### RNA extraction, cDNA synthesis and quantitative real-time PCR

Total RNA previously used for RNA-Seq was used for the construction of cDNA. Total RNA that was previously used for RNA-Seq was extracted by Trizol method following the manufacturer’s protocol followed by cDNA synthesis. circRNAs were enriched in the total RNA pool by using RNA exoribonucleases (RNase R).The circRNA enriched samples with depleted ribosomal RNA were reverse transcribed into cDNA with random hexamer primers using iScript RT Supermix Kit (Bio-Rad) following the manufacturer’s procedure. cDNA synthesized using specific divergent sets of primers were subjected to RT-qPCR. Expermient was performed in triplicate for RT-qPCR. A total reaction of 20 µl was made with 0.5 µl (10 µM) of each forward and backward divergent primer, 10 µl of SYBR Green PCR master mix (Roche), 1 µl (70 ng/  µl) of cDNA and 8 µl of nuclease free water. Glyceraldehyde-3-Phosphate (GAPDH) was used as an internal control. The experimental validation was selected for the group of five circRNAs, including circ_03409, circ_87295, circ_03409a, circ_10119b and circ_25279. For the selected circRNAs, five divergent primers were designed using Primer3 Plus software. The details of the primers are given in (Table S1). The relative quantification was determined using the 2^−ΔΔ*Ct*^ method (Livak and Schmittgen, 2001).

### Functional enrichment analysis

To gain insight about the function of mRNAs encoded by similar DE circRNAs, we performed Gene Ontology (GO) and pathways enrichment analysis. The enrichment analysis was performed utilizing KOBAS 3 server (Wu et al., 2006). *Bos taurus* gene-set were used as background (Bhat et al., 2019a; Bhat et al., 2019b).

## Results

**Figure 1:**
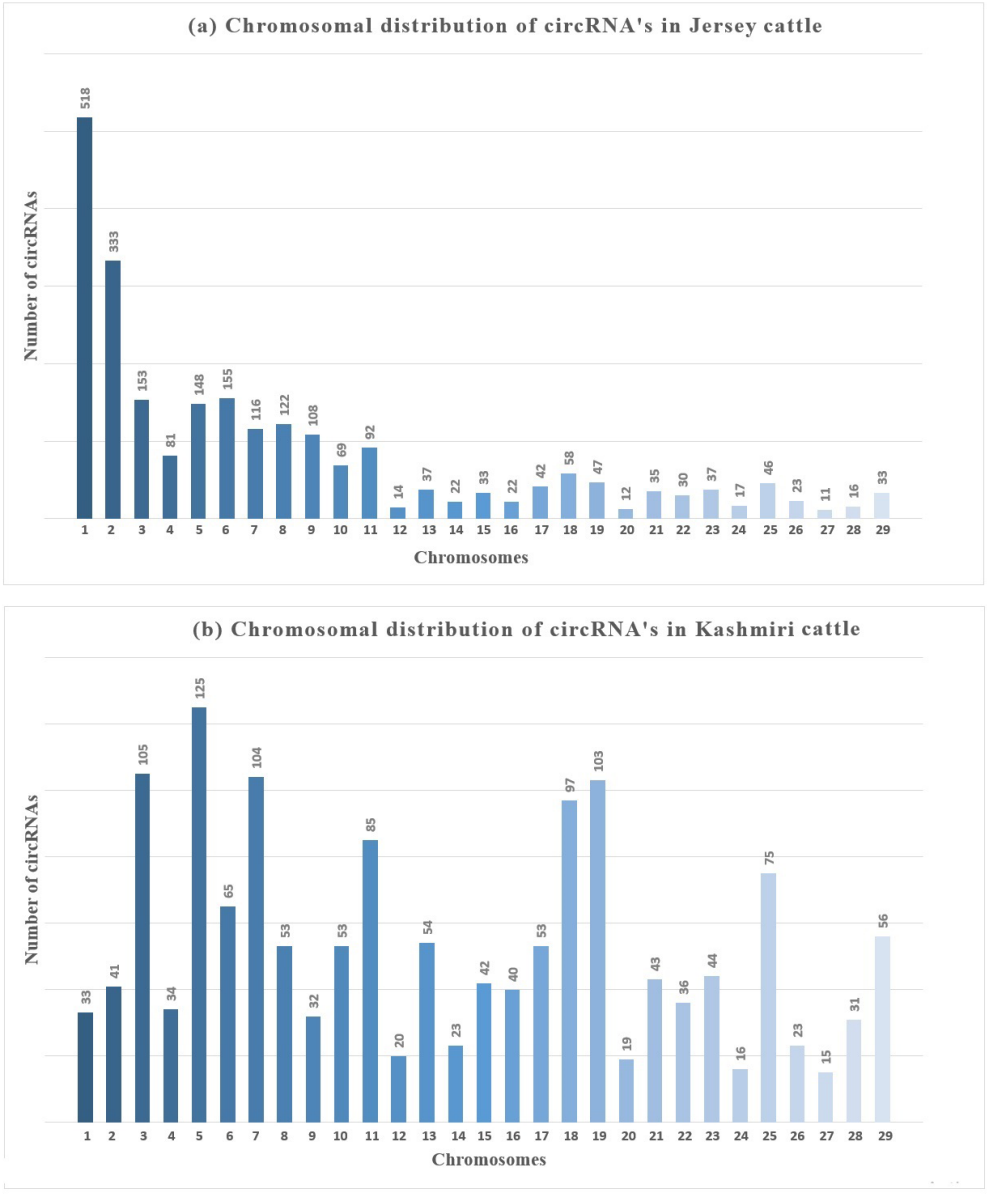
Chromosomal distribution of circRNAs in mammay gland epithelial cells of (A) Jersey and (B) Kashmiri cattle at peak lactation.

An average of 2986 circRNAs were reported from the samples analyzed, majority of which were ecircRNAs (≈ 72.16%/2154) whereas ciRNAs were less common (≈ 27.83%/831). Most of the circRNAs were distributed on chromosome (chr) 5 (≈ 20.26% /605 and chr 6 (≈7.23%/ 216) while fewest circRNA were distributed on chr 12, 24, 26 and 27. The number of circRNAs obtained from mammary glands of Jersey and Kashmiri were 1554 (966 exonic, 588 intronic) and 1286 (1047 exonic, 239 intronic) respectively. In Jersey, a higher number of circRNAs were derived from chr 4 (125 circRNAs) followed by chr 3 (105 circRNAs), chr 7 (104) and chr 19 (103). While as in Kashmiri cattle 518 circRNAs were derived from chr 1 followed by chr 2 (333), chr 6 (155), chr 3 (153) and chr 7 (Fig. 1).

The most abundant and highly expressed circRNAs in both Kashmiri and Jersey cattle were circ“-_003409, circ_010119, circ_081923, circ_006590, circ_021268, circ”-_023981 and circ_013402 which were derived from CSN2, CSN1S1, PAEP, CSN1S2, RPS5, NUPR1 and TPT1 respectively. A total of 150 circRNAs were obtained from casein protein that includes 87 CSN1S1, 31 CSN1S2, 29 CSN2, and 03 CSN3 circRNAs. Two circRNAs, LALBA and BLG were derived from whey protein coding genes.

### Analysis of differentially expressed circRNAs

There were 21 differentially expressed circRNAs between Kashmiri and Jersey breeds with log2FC >1 and *P*-value <0.05, with 14 up-regulated circRNAs in Kashmiri cattle and 7 up-regulated circRNAs in Jersey. All differentially expressed circRNAs were found to be exonic, only one circ_60634 derived from RHBDD2 gene (Start: 34128469, Stop: 34128556, Strand: +, Exon count: 01) present on chr 25 and upregulated in Jersey breed was intronic. The DE circRNA were compared against the known circRNAs from *Homo sapiens, Mus musculus, Sus scrofa, Gallus gallus* and *Canis lupus*. A total of 8 out of 21 DE circRNAs were mapped with the threshold of e-value <0.00001 and similarity >85% (Table S2). Seven DE circRNAd were derived from casein genes (03 circRNAs from CSN2 gene, 03 circRNAs from CSN1S1, 01 circRNA from CSN1S2) (Table S3). The circRNAs that were upregulated in Kashmiri cattle included circ_03409 (gene CSN2 on chr 6), circ_03409A (gene CSN2 on chr 6), circ_03409B (gene CSN2 on chr 6),circ“-_81923A (gene PAEP on chr 11), circ”-_81923B (gene PAEP on chr 11) circ_10119A (gene CSN1S1 on chr 6), circ_10119B (gene CSN1S1 on chr 6), circ_10119C (gene CSN1S1 on chr 6) circ_63604 (gene CSN1S1 on chr 25), circ_06590 (gene CSN1S2 on chr 6), circ_21268 (gene RPS5 on chr 8), circ_61308 (gene ATRX on chr X), circ_81923 (gene PAEP on chr 11), and circ_70874 (gene PICALM on chr 29). CircRNAs upregulated in Jersey breed with respect to Kashmiri cattle include circ_87295 (gene USP3 on chr 10), circ_23981 (gene NUPR1 on chr 25), circ_82741(gene NDEL1 on chr 19), circ_13402 (gene TPT1 on chr 12), circ_25279 (gene ITGB7 on chr 5), circ_20009 (gene CD14 on chr 7) and circ_34753 (gene KANSL1 on chr 19). Among these DE circRNAs the three highly abundant circRNAs upregulated in Kashmiri cattle were: circ_81923A, circ_03409A and circ_10119B. Most abundant circRNAs upregulated in Jersey breed with respect to Kashmiri cattle include circ_87295 and circ_25279. To confirm the authenticity of the differentially expressed circRNAs, we performed RT-qPCR of only five circRNAs *i.e.,* 03 DE circRNAs from Kashmiri cattle (circ_03409, circ_03409A and circ_10119B) and 02 DE circRNAs from jersey cattle (circ_87295 and circ_25279). The different suffixes indicate the circRNAs derived from same genes. The findings demonstrated that the head-to-tail backsplicing of circRNAs was consistent with results of RNA-Seq, verifying the reliability and integrity of the circRNAs discovered in the analysis.

### Enrichment analysis of differentially expressed genes

We analyzed the cluster analysis of gene ontology (GO) and KEGG pathway enrichment of differentially expressed circRNAs to further investigate the relationship between circRNAs and regulation of gene expression. The parental genes for 21 differentially expressed circRNAs were primarily enriched in 09 biological processes including response to hormones like progesterone, estradiol, dehydroepiandrosterone,11- deoxycorticosterone and growth hormone, response to lipids, drugs, cellular oxidant detoxification, 01 molecular function showing antioxidant activity and 05 cellular components showing strong enrichment for terms related to golgi lumen, extracellular region, golgi apparatus, extracellular space and endomembrane system. The KEGG pathway revealed that the developmental genes of circRNAs were concentrated in a variety of pathways including intestinal immune network for IgA production, pertussis pathway, prolactin pathway, salmonella infection, Toll like receptor pathway, ECM receptor interaction, MAPK signalling pathway and PI3K-Akt signaling pathway, NF kappa B signalling pathway, and cell adhesion molecules (Tables S4 and S5).

### Validation of identified circRNAs by Real Time-qPCR

**Figure 2:**
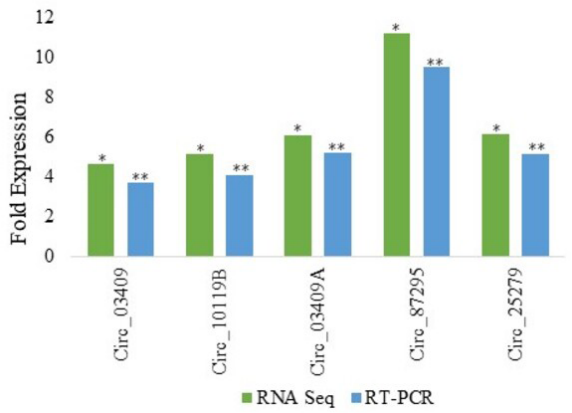
Real time PCR validation of differentially expressed circRNAs in Jersey and Kashmiri cattle. The y-axis represents the log2 fold change of circRNAs expression; the x-axis shows the circRNA IDs employed for validation. RNA seq:*P < 0.05; RT-qPCR: **P < 0.05. Suffixes A and B represent circRNAs derived same gene having same ensemble ID.

Among various DE circRNAs, RT-qPCR of five differentially expressed circRNA from Kashmiri and Jersey breed was performed to confirm the reliability as well as accuracy and reliability of RNA-Seq data. From Kashmiri cattle we selected 03 DE circRNAs (circ_03409, circ_03409A and circ_10119B and in case of Jersey cattle circ_25279 and circ_87295 were selected for validation of RNA-Seq results. As already mentioned above,the different suffixes indicate the circRNAs derived from same genes. The results of RT-qPCR revealed that the levels of expression of DE circRNAs and RNA-Seq are highly accurate and precise (Fig. 2).

## Discussion

Hormones, growth factors, and the availability of nutrients closely control the production and functional initiation of lactation in the mammary gland. Previous studies have concluded that mammary glands milk production and lactation success in mammals is influenced by a substantial number of ncRNAs and various functional genes (Miglior et al., 2017; Wang et al., 2012). Analysis of the molecular mechanism involved in mammary gland development and lactation is thus essential for promoting milk yield and quality in livestock. CircRNAs are stable molecules as they lack free 3′ and 5′ ends. Therefore, in the context of circRNAs function in mammary glands, we studied the circRNAs expression levels in two dairy cattle breeds viz Kashmiri and Jersey, to investigate how circRNAs play a role in controlling the expression of milk-related genes.

Previous studies suggest that in bovine MECs, circular RNAs were abundant, and their levels of expression differed at various lactation stages (Zhang et al., 2016a; Zhang et al., 2016b). CircRNAs function as sponges of miR-2284 that control the translation of casein genes and thus regulate the synthesis of milk protein (Zhang et al., 2016a; Zhang et al., 2016b). The disparity in the production of circRNA between species may be due to their different lactating periods. In our study, 1554 and 1286 circRNAs were obtained at peak lactation in Jersey and Kashmiri cattle respectively. The majority of circRNAs *i.e.,* 1780 originated from multiple exons, which accounted for 59.63%, and 1205 were found to be single exonic. Similar results were obtained from mammary gland of sheep, where the majority of circRNAs originated from multiple exons (Li et al., 2015). A total of 150 circRNAs were produced from 7 casein genes and 2 from whey protein genes. The most abundant circRNAs were derived from alpha s1-casein gene (CSN1S1). These results were similar to the previous studies in sheep (Hao et al., 2020a; Hao et al., 2020b) and bovine (Memczak et al., 2013). Studies have found that high milk yield and protein content were correlated with the CSN1S1 gene (Van Eenennaam & Medrano, 1991). Beta-casein gene (CSN2) is essential for the structure of casein micelle and high milk yield (Zwierzchowski, 2005; Jann et al., 2002). A milk protein that is encoded by kappa casein gene (CSN3) is essential for the formation, stability and structural characteristics of casein micelles and is a key protein in the development of cheese (Bingham & Groves, 1979). In addition to this, LALBA gene codes for alpha-lactalbumin protein which functions as lactose synthases essential regulatory subunit, in mammary gland alters the substrate specificity of galactosyltranferase, rendering glucose a suitable acceptor substrate for this galactosyltranferase enzyme. In the current study, we found two circRNAs, LALBA and BLG were derived from whey protein coding genes. Findings from all these previous studies suggest that the circRNAs obtained from these casein and whey genes may be involved in enhancing the milk production and quality in bovines. Further, these circRNAs may also be associated with growth and development of mammary gland but this presumption requires further verification.

In an attempt to further characterize the biological processes of circRNAs that are differentially expressed in two breeds, GO and KEGG enrichment pathway analysis of host genes of DE circRNAs was performed. The differentially expressed circRNAs showed response to progesterone, estradiol, growth hormone, dihydroxyepiandosterone (DHEA), deoxycorticosterone, oxygen containing compounds, drug response and cellular oxygen detoxification. Previous studies have already shown that progesterone alone with prolactin has a role in generating milk alveoli cells which secrete milk during lactation (Macias & Hinck, 2012). Further studies using microarray assays revealed 124 genes in mammary glands of mammals whose transcription is controlled by oestrogen, the majority of which are associated with cell multiplication and proliferation activation like epidermal growth factor 1 (EGF1), cyclin D1 and insulin growth factor-1 (IGF-1) (Connor et al., 2007). Some studies have also shown that estradiol is capable of regulating transcription of progesterone receptor (Petz et al., 2004). Previous research has shown that progesterone binds to its receptor in the mammary glands to cause Wnt-4 gene transcription, thus mediating role of progesterone in cell proliferation (Brisken & O’Malley, 2010). Two steroid hormones, DHEA and deoxycorticosterone, on the other hand, serve in synthesis of milk. The parental genes of DE circRNAs also showed strong enrichment of antioxidant activity. Golgi lumen, extracellular region, golgi apparatus, extracellular space and endomembrane system were top enriched GO terms of parental genes. This might be because milk proteins are synthesized in endoplasmic reticulum and processed in golgi apparatus where these are released in vesicles before secretion.

The host genes of circRNAs were abundantly expressed in the prolactin pathway, Ras, Mitogen Activated Protein Kinase signalling pathways (MAPK), Phosphatidylinositol 3-Kinase (PI3K) and Akt Kinase signalling pathway in KEGG pathway enrichment. In the epithelial cells of mammary gland, the PI3K-Akt signalling pathway as well as MAPK through Stat5 and mTOR and also prolactin-mediated activation of the Jak2-Stat5 pathway, regulate the majority of metabolic mechanisms and biological pathways including transcriptional activation of genes for proliferation as well as cell cycle and synthesis of milk protein (DaSilva et al., 1996; Liu et al., 1997; Chen et al., 2012; Bhat et al., 2017). Other substantially enriched pathways including the NF- *κ*B signalling pathway, the intestinal immune network for IgA development and production and Toll-like receptor pathway were related to immunity. On the basis of the study done, circRNAs identified in our study might have the capability to act as regulators of milk synthesis and stimulate growth and development of mammary glands.

AMPK plays an essential role in cellular energy sensing (Hardie, Ross & Hawley, 2012) and mTOR activation (Inoki, Zhu & KL, 2003). This signaling pathway is found to be involved in regulating the effect of glucose supply and utilization in the lactating mammary glands (Bionaz, Hurley & Loor, 2012). It is well known that mTOR is an essential regulator in milk protein synthesis, and most studies are concerned with the role of AAs in the regulation of P-mTOR on Ser^2448^ in milk protein synthesis (Appuhamy et al., 2012; Apelo, Knapp & Hanigan, 2014). In the present study 150 circRNAs were obtained from casein protein only.

In our previous study (Ahmad et al., 2021) we found in Jersey cattle, the phosphatidylinositol 3-kinase (PI3K)/protein kinase B (Akt) signaling pathway was most enriched. PI3K-Akt pathway is important for normal insulin-mediated glucose metabolism (Taniguchi et al., 2006). Jersey being higher milk producer breed requires more energy source than Kashmiri cattle. Toll like receptor 4 (TLR4) signaling results in the activation of NF- *κ*B pathway and releases pro-inflammatory cytokines (Medzhitov & Kagan, 2006). NF- *κ*B being an essential transcriptional factor, regulates immune response and gene expression of many inflammatory cytokines (Putra et al., 2014). As supported by our earlier findings most of the immune related pathways were found exclusively expressed in Kashmiri cattle (Bhat et al., 2019a; Bhat et al., 2019b; Bhat et al., 2020; Ahmad et al., 2021). This suggests the possible defense mechanism of Kashmiri cattle against mammary gland infections. Based on our results and data from recent studies, circRNAs have been found to have a significant role in milk synthesis and in the development of the mammary gland in cattle.

Although numerous studies have reported that the circRNAs are tissue-specific and conserved across different species (Rybak-Wolf et al., 2015; Chen, 2016; Mumtaz et al., 2020). In the present study we demonstrated that the DE circRNAs showed significant similarity (>85%) across various species like *Homo sapiens* and *Sus scrofa*. The target gene analysis of DE circRNs in cattle were 100% similar for UP3 and PICALM genes in *Sus scrofa* and RPS5 gene in *Homo sapiens*.

## Conclusion

The function of circRNAs remains largely unclear in the lactation physiology of dairy animals. In the present study an association between circRNAs and milk synthesis has been reported using RNA-seq data. The results suggest that circRNAs are abundant in bovine MECs and the expression level of circRNAs varied between Jersey and Kashmiri cattle. The number of circRNAs obtained from MECs of Jersey were high (1,554) compared to Kashmiri cattle (1,047). The results also reveal that among the total circRNAs identified, 21 circRNAs were expressed differentially between Jersey and Kashmiri cattle. Most of the DE circRNAs are derived from casein genes. This signifies the role of circRNAs in lactation of animals. The chromosomal distrubition revealed most of circRNAs were assembled on chr 4 in Jersey, while as in Kashmiri cattle most circRNAs were gathered on chr 1. These results suggest breed specific distribution and variation of circRNAs in cattle. The pathway analysis shows that most enriched pathways like MAPK, IP3K and prolactin pathway are involved in milk synthesis. These findings add to our understanding of the functional role of circRNAs in the production of bovine milk. Further anlysis revealed that identified circRNAs were highly conserved across various species like *Homo sapiens, Sus scrofa and Canis lupus.*

## Supplemental Information

10.7717/peerj.13029/supp-1 Supplemental Information 1 CircRNAs and primer sequences used for validation of RNA-Seq data

10.7717/peerj.13029/supp-2 Supplemental Information 2 Comparative analysis of the CircRNA with known circRNAs

10.7717/peerj.13029/supp-3 Supplemental Information 3 DE circRNAs between Kashmiri and Jersey cow breeds, their chromosomal location, exon count and size, log2 fold expression, genes with which CircRNAs are related and *P*-values

10.7717/peerj.13029/supp-4 Supplemental Information 4 The DE CircRNAs between Kashmiri and Jersey cow breeds, different biological, molecular and cellular functions with which these circRNAs are associated

10.7717/peerj.13029/supp-5 Supplemental Information 5 The gene ontology and KEGG analysis of the DE circRNAs between Kashmiri and Jersey cow breeds

10.7717/peerj.13029/supp-6 Supplemental Information 6 Normalisation of housekeeping gene GAPDH in Kashmiri and Jersey cattle breeds

10.7717/peerj.13029/supp-7 Supplemental Information 7 Quantitative real time PCR efficiency of primers used in the study

## Abbreviations

CircRNA: circular RNA
ncRNA: non-coding RNA
MEC’s: mammary epithelial cells
MAPK: Mitogen-activated protein kinases
IP3K: Phosphatidylinositol-3-Kinase
CSN 2: beta- casein
CSN1S1: casein alpha s1
protein (PAEP): progestogen-associated endometrial
*CSN1S2*: alpha-S2 casein *gene*
RPS5: Ribosomal Protein S5
NUPR1: Nuclear protein 1
TPT1: Translationally Controlled Tumor
LALBA: Lactalbumin-alpha
BLG: beta-lactoglobulin
*RHBDD2*: Rhomboid domain-containing protein 2
USP3: Ubiquitin Specific Peptidase 3
NDEL1: Nuclear distribution protein nudE-like 1
ITGB7: integrin subunit beta 7
KANSL1: KAT8 Regulatory NSL Complex Subunit 1
ECM: extracellular matrix
*DHEA*: dehydroepiandrosterone

## Additional Information and Declarations

### Data Availability

The following information was supplied regarding data availability: The sequences are available at NCBI GEO: SRR6324372.
