# Supplementary material for: Genome wide expression analysis of circular RNAs in mammary epithelial cells of cattle revealed difference in milk synthesis
Source: PeerJ. 2022 Mar 1;10:e13029. doi: 10.7717/peerj.13029 (PMC8896013; doi:10.7717/peerj.13029)
Supplement: Supplemental Information 1 [file peerj-10-13029-s001.docx]

| **CircRNAs** | **Primer sequence (5′ → 3′)** | | **Annealing**  **T_m °C_** | **Size of Amplicon** |
| --- | --- | --- | --- | --- |
| Circ_03409 | Forward primer | TGTTTGCTGCTGTTCCTCAC | 59.6 | 160 |
|  | Reverse primer | GTGGGTTGTCATCTCCTCCA |  |  |
| *CIRC_87295* | Forward primer | CTGTGTGCTGTGCTTTTTCTT | 57.3 | 108 |
|  | Reverse primer | TTTCTCCAAGGTATGTGAATGG |  |  |
| *Circ_03409a* | Forward primer | GTGCTGAGGTTCTGCTGAGG | 58.25 | 107 |
|  | Reverse primer | CCATTCATTCATGTTTCTCTCTTC |  |  |
| *Circ_10119b* | Forward primer | CCATTCATTCATGTTTCTCTCTTC | 56.45 | 120 |
|  | Reverse primer | AATTTTTCTCTTTAATCCCTCAGTT |  |  |
| *Circ_25279* | Forward primer | ATCATTGTGCTGGGCTGTGT | 60.35 | 128 |
|  | Reverse primer | AGATGCTGCCGCTCCTTT |  |  |
| *GAPDH* | Forward | GCAAGTTCCACGGCACAG | 65 | 213 |
|  | Reverse | GGTTCACGCCCATCACAA |  |  |

**Supplementary Table S1.** CircRNAs and primer sequences used for validation of RNA-Seq data by qPCR.
