# Supplementary material for: Genome wide expression analysis of circular RNAs in mammary epithelial cells of cattle revealed difference in milk synthesis
Source: PeerJ. 2022 Mar 1;10:e13029. doi: 10.7717/peerj.13029 (PMC8896013; doi:10.7717/peerj.13029)
Supplement: Supplemental Information 2 [file peerj-10-13029-s002.docx]

| **Query_SeqID** | **Subject_SeqID** | **Organism** | **Target genes** | **Sequence Similarity** | **E-Value** |
| --- | --- | --- | --- | --- | --- |
| *10:46480908-46485029* | sus-USP3_0001 | *Sus scrofa* | USP3 | 100 | 2.26E-40 |
| *X:74734797-74749815* | hsa-ATRX_0023 | *Homo sapiens* | ATRX | 94.444 | 1.17E-05 |
| *19:46134505-46135877* | can-KANSL1_0030 | *Canis lupus* | KANSL1 | 86.957 | 1.91E-33 |
| *25:26082677-26082824* | hsa-NUPR1_0001 | *Homo sapiens* | NUPR1 | 87.5 | 2.02E-21 |
| *12:15474144-15474230* | sus-ENSSSCG00000034878_0001 | *Sus scrofa* | ENSSSCG00000034878 | 92.857 | 8.88E-13 |
| *19:28026804-28046307* | sus-NDEL1_0013 | *Sus scrofa* | NDEL1 | 98.276 | 1.98E-168 |
| *18:65661037-65661146* | hsa-RPS5_0008 | *Homo sapiens* | RPS5 | 100 | 6.16E-18 |
| *29:9552574-9605079* | sus-PICALM_0031 | *Sus scrofa* | PICALM | 100 | 0 |

**Supplementary Table S2**. Comparative analysis of the CircRNA with known circRNAs.
