# Supplementary material for: Genome wide expression analysis of circular RNAs in mammary epithelial cells of cattle revealed difference in milk synthesis
Source: PeerJ. 2022 Mar 1;10:e13029. doi: 10.7717/peerj.13029 (PMC8896013; doi:10.7717/peerj.13029)
Supplement: Supplemental Information 6 [file peerj-10-13029-s006.pdf]

GAPDH KASHMIRI

Mean	15.6
Standard Error	0.044
Median	15.63
Mode	15.38
Standard Deviation	0.153
Sample Variance	0.024
Kurtosis	-0.439
Skewness	0.01
Range	0.51
Minimum	15.38
Maximum	15.89
Sum	187.3
Count	12

GAPDH JERSEY

Mean	15.65
Standard Error	0.04
Median	15.63
Mode	15.45
Standard Deviation	0.141
Sample Variance	0.02
Kurtosis	0.698
Skewness	0.862
Range	0.5
Minimum	15.45
Maximum	15.95
Sum	187.86
Count	12

Group	ID	CT1	CT2	Avg	Stdev	SEM
Jersey	C1J	15.55	15.65	15.65	0.141	0.04
	C2J	15.45	15.62			
	C3J	15.58	15.67			
	C4J	15.61	15.51			
	C5J	15.69	15.71			
	C6J	15.87	15.95			

GAPDH JERSEY

Mean	15.65
Standard Error	0.04
Median	15.63
Mode	15.45
Standard Deviatioı	0.141
Sample Variance	0.02
Kurtosis	0.698
Skewness	0.862
Range	0.5
Minimum	15.45
Maximum	15.95
Sum	187.86
Count	12
Confidence Level(	0.314323

Group	ID	CT1	CT2	Avg	Stdev	SEM
	C1K	15.62	15.65			
	C2K	15.89	15.75			
Kashmiri	C3K	15.72	15.69	15.6		
	C4K	15.38	15.49		0.153	0.044
	C5K	15.38	15.49			
	C6K	15.56	15.68			

GAPDH KASHMIRI

Mean	15.6
Standard Error	0.044
Median	15.63
Mode	15.38
Standard Deviati	0.153
Sample Variance	0.024
Kurtosis	-0.439
Skewness	0.01
Range	0.51
Minimum	15.38
Maximum	15.89
Sum	187.3
Count	12
Confidence Leve	0.757164
