# Supplementary material for: Genome wide expression analysis of circular RNAs in mammary epithelial cells of cattle revealed difference in milk synthesis
Source: PeerJ. 2022 Mar 1;10:e13029. doi: 10.7717/peerj.13029 (PMC8896013; doi:10.7717/peerj.13029)
Supplement: Supplemental Information 7 [file peerj-10-13029-s007.pdf]

PCR efficiency and standard curve GAPDH

dilution	cDNA a	ln cDN.	CT
1x	1	0	13.21
2x	0.333	-1.1	14.15
4x	0.2	-1.61	14.93
8x	0.111	-2.2	16.31
16x	0.059	-2.83	16.74
32x	0.03	-3.5	17.54
64x	0.015	-4.17	20.18
linear regr. of C		k	-1.58
		d	12.68
		y = kx + d	
slope (calc. for 10x dil.)			-1.09
PCR efficiency			1.886

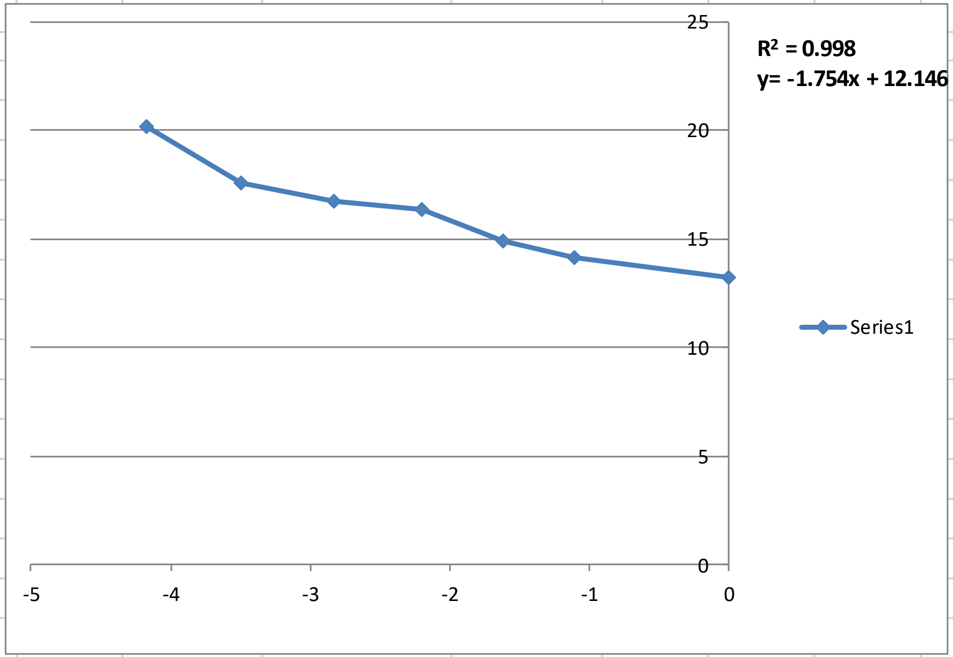

SUMMARY OUTPUT

Regression Statistics	
Multiple	0.999
R Squa	0.998
Adjuste	0.998
Standa	0.13
Observ	4

ANOVA

	df	*SS*	*MS*	*F*	*gnificance F*
Regres	1	21.49	21.4861	1279.2925	0.00078
Residu	2	0.034	0.0168		
Total	3	21.52			

	Coefficien	*ndard E*	*t Stat*	*P-value*	*.ower 95%*	*pper 95*	*wer 95.(*	*per 95.0%*
Interce	11.88	0.142	83.7951	0.0001424	11.2714	12.49	11.27	12.49
0	-1.99	0.056	-35.767	0.0007808	-2.22635	-1.75	-2.23	-1.75

RESIDUAL OUTPUT

Observati	*licted 1*	*esiduals*
1	14.06	0.085
2	15.08	-0.15
3	16.25	0.062
4	20.18	0.003

PCR efficiency and standard curve of Circ_25279

dilution	cDNA amount	ln cDNA	CT
1x	1	0	22.67
2x	0.333333	-1.09861	24.5
4x	0.2	-1.60944	24.97
8x	0.111111	-2.19722	26.31
16x	0.058824	-2.83321	26.98
32x	0.030303	-3.49651	28.18
64x	0.015385	-4.17439	30.87
linear regr. of Ct / ln(DNA):		k	-1.54536
		d	22.69424
		y = kx + d	
	slope (calc. for 10x dil.)		-1.07116
	**PCR efficiency**		**1.909988**

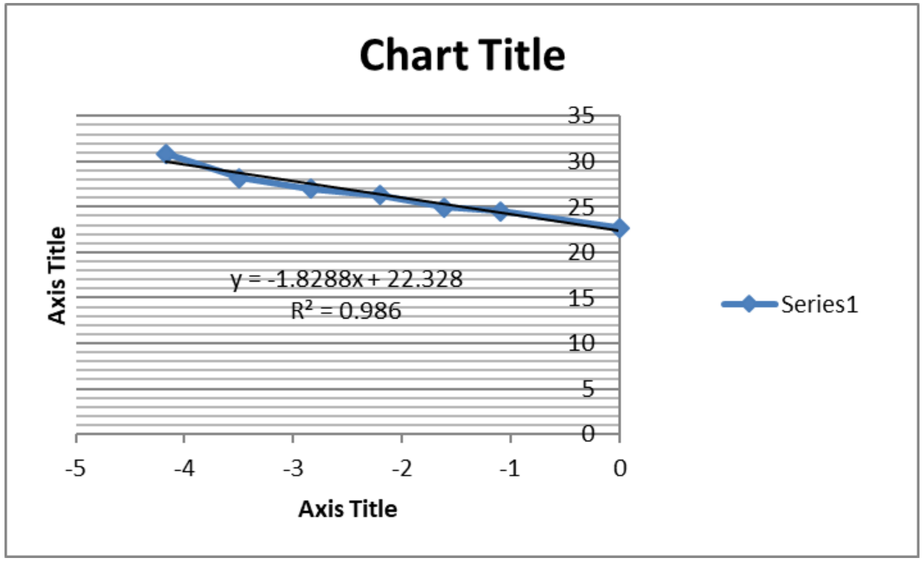

SUMMARY OUTPUT

Regression Statistics	
Multiple R	0.99342
**R Square**	**0.986883**
Adjusted R Square	0.982511
Standard Error	0.197742
Observations	5

ANOVA

	df	*SS*	*MS*	*F*	*Significance F*
Regression	1	8.825774	8.825774	225.7124	0.00064
Residual	3	0.117306	0.039102		
Total	4	8.94308			

	Coefficients	*Standard Error*	*t Stat*	*P-value*	*Lower 95%*	*Upper 95%*	*Lower 95.0%*	*Upper 95.0%*
Intercept	22.6856	0.249334	90.9847	2.93E-06	21.8921	23.47909	21.8921	23.47909
0	-1.5587	0.103749	-15.0237	0.00064	-1.88888	-1.22853	-1.88888	-1.22853

RESIDUAL OUTPUT

Observation	*Predicted 22.67*	*Residuals*
1	24.39801	0.101994
2	25.19423	-0.22423
3	26.11042	0.199584
4	27.10173	-0.12173
5	28.13561	0.044388

PCR efficiency and standard curve of Circ 03409

**dilution**	**cDNA amount**	**ln cDNA**	**CT**
1x	1	0	28.88
2x	0.333333	-1.09861	30.94
4x	0.2	-1.60944	31.73
8x	0.111111	-2.19722	32.32
16x	0.058824	-2.83321	33.23
32x	0.030303	-3.49651	36.88
64x	0.015385	-4.17439	
linear regr. of Ct / ln(DNA):		k	-1.51375
		d	29.07718
		y = kx + d	
	slope (calc. for 10x dil.)		-1.04925
	**PCR efficiency**		**1.935978**

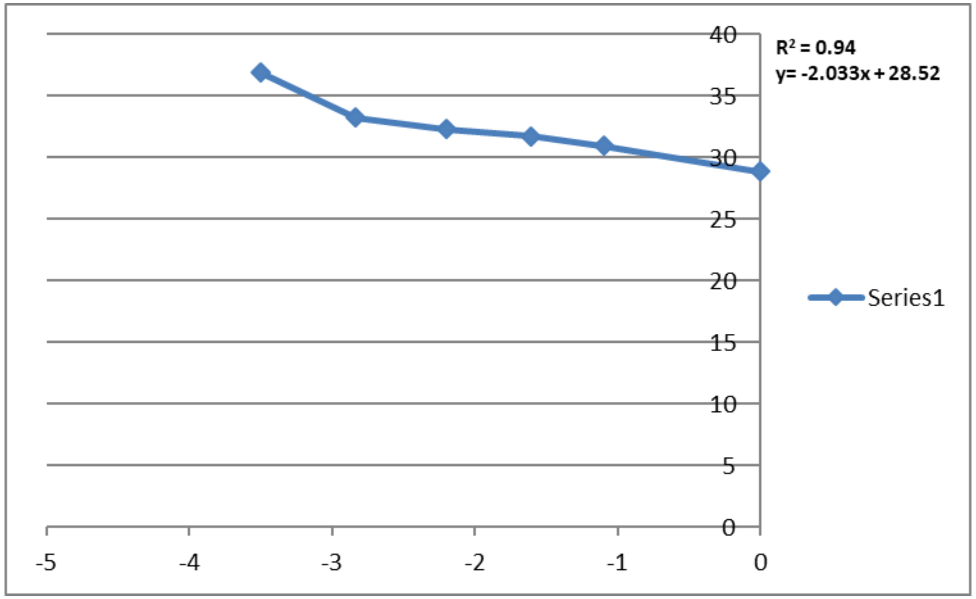

SUMMARY OUTPUT

Regression Statistics	
Multiple R	0.99715
**R Square**	**0.943076**
Adjusted R Square	0.991461
Standard Error	0.089267
Observations	4

ANOVA

	df	*SS*	*MS*	*F*	*Significance F*
Regression	1	2.783763	2.783763	349.345	0.00285
Residual	2	0.015937	0.007969		
Total	3	2.7997			

	Coefficients	*Standard Error*	*t Stat*	*P-value*	*Lower 95%*	*Upper 95%*	*Lower 95.0%*	*Upper 95.0%*
Intercept	29.56547	0.140475	210.4678	2.26E-05	28.96105	30.16988	28.96105	30.16988
0	-1.28683	0.068848	-18.6908	0.00285	-1.58306	-0.9906	-1.58306	-0.9906

RESIDUAL OUTPUT

Observation	*Predicted 28.88*	*Residuals*
1	30.9792	-0.0392
2	31.63654	0.093457
3	32.39293	-0.07293
4	33.21134	0.018665
